# Evaluation of the outcomes of vagal nerve stimulation in children with drug-refractory epilepsy in South Africa

**DOI:** 10.1007/s00381-025-07110-x

**Published:** 2026-01-17

**Authors:** Edmund Kibuuka, Denis Mutyaba, John Ouma, Jason Labuschagne

**Affiliations:** 1 Department of Neurosurgery, University of the Witwatersrand, Johannesburg, South Africa; 2 Department of Paediatric Neurosurgery, Nelson Mandela Children’s Hospital, Johannesburg, South Africa; 3 Nelson Mandela Children’s Hospital, 6 Jubilee Road, Parktown, Johannesburg, 2193 South Africa

**Keywords:** Vagal nerve stimulation, LMIC, Neuromodulation

## Abstract

**Purpose:**

To evaluate the clinical outcomes of vagal nerve stimulation (VNS) in children with drug refractory epilepsy (DRE) in a Sub-Saharan setting.

**Methods:**

We retrospectively reviewed the data of 93 paediatric patients with DRE who underwent VNS insertion between January 2008 and December 2022. Patient demographics, aetiology of epilepsy and seizure data were analyzed. Seizure outcomes were measured using the Engel, McHugh, and Hague scores. Statistical analysis was performed using the Chi-square test, T-tests, and Spearman’s correlation.

**Results:**

Of the 93 patients who underwent VNS implantation 60% achieved a seizure reduction of at least 50%. Demographic factors associated with improved seizure control included age at implantation (*p* < 0.001) and shorter duration from first reported seizure to implantation (*p* < 0.001). The mean time to response was 6.9 months. The McHugh scale showed a significant association between the length of follow up and VNS response. Adverse effects of VNS insertion were limited. Our outcome data and complication rates were in line with previous studies from the developed world. Challenges unique to our population included delay from diagnosis to referral; on average, there was a 5-year period from diagnosis of DRE to referral to our center. In addition to the large population and our extensive geographic referral pattern, we identified that a key factor in delayed referral to our center was due to a poor awareness, amongst both caregivers and referring clinicians of VNS as a treatment option for DRE.

**Conclusion:**

VNS is a safe and effective adjunctive treatment for children with DRE in the Sub-Saharan population, with results comparable to centers in the developed world. In our cohort of patients, the McHugh scale appeared to be a more sensitive tool for assessing and tracking VNS response.

## Introduction

Vagal nerve stimulation (VNS) is a well-established treatment modality for drug-refractory epilepsy (DRE), which is not amenable to epilepsy surgery in the paediatric and adult populations, with the literature reporting efficacy rates in the region of 60% [9, 12, 18, 25].

Although its efficacy and safety have been studied in the paediatric population in the developed world setting, there is far less literature on its use in Sub-Saharan Africa. We aimed to expand on the current literature regarding the use of VNS in a paediatric population with DRE particularly regarding the demographic differences and if any clinical characteristics were associated with improved outcomes post-VNS insertion. To the best of our knowledge, this is the first study to assess the efficacy and safety profile of VNS in the Sub-Saharan setting.

## Methods

### Study population

This retrospective study reviewed the data of 93 patients between the ages of 6 months and 12 years old with drug-refractory epilepsy who underwent VNS insertion by a single surgeon (J.L.) between January 2008 and December 2022. All the cases were preselected by an epileptologist and presented at a multi-disciplinary epilepsy surgery conference. Assessment was based on clinical semiology, MRI findings and surface EEG recordings. Less than 10% of the cohort had undergone invasive electrophysiological monitoring. The typical candidate for VNS therapy either had no identifiable lesion on MRI, had divergent preoperative data with respect to the clinical semiology, EEG and structural imaging or had a lesion encroaching on eloquent cortex. Likewise, children with DRE with widespread changes on MRI not amenable to surgery or non-localizing/ambiguous seizure origin were considered candidates for VNS therapy. VNS therapy was offered for both focal and generalized seizure or epilepsy type. Syndromic epilepsy and metabolic etiologies for the epilepsy were not considered a contraindication to the procedure.

The minimum follow-up period was 12 months. Data collection forms were utilized to capture the patient’s history, demographics, aetiology of epilepsy and epilepsy syndrome using the ILAE classification, predominant seizure type using the ILAE classification, previous antiepileptic drug treatment, type of VNS device inserted, and device and surgery-related complications.

### Follow-ups

Seizure frequency and severity was assessed prior to VNS insertion, within 90 days post-VNS insertion, and at each follow up visit. Responder status was defined as a reduction of seizure frequency of at least 50%. Previously validated models, including the Engel Epilepsy Outcome Scale [7], the McHugh Seizure Reduction Score [14], and the Hague Seizure Severity Scale [4], were used to grade changes in seizure frequency and severity.

The Engel scale is a scale used to assess the outcome of epilepsy surgery. It consists of four classes, 1–4, which represent the best outcome (Class 1: Seizure freedom) to the worst outcome (Class 4: No worthwhile improvement) [7].

The McHugh scale is a scale used to assess the efficacy of VNS treatment for epilepsy. It consists of 5 classes, which represent the best outcome (Class 1: 80 −100% reduction in seizure frequency) to the worst outcome (Class 5: No improvement in seizure frequency) [14], see Table 3.

The Hague Seizure Severity Score is a subjective questionnaire completed by caregivers based on their perception of their child's seizure activity and severity. It assesses seizure severity and its impact on daily life. Scores range from 13–54, with lower scores indicating fewer disabling seizures and higher scores indicating more disabling seizures [4].

Follow up data was stratified into six intervals: at 12 months; 13–17 months; 18 months; 19- 23 months; 24 months; and greater than 24 months and assessed according to the above-mentioned validated scales.

### Surgery

VNS implantation was performed by a single surgeon (J.L.) according to standard procedures. All VNS devices were implanted on the left. Three different VNS Therapy® devices were used in the sample namely, The Pulse® 102 (LivaNova, Houston, TX, USA), AspireSR® 106 (LivaNova, Houston, TX, USA) and the Sentiva® 1000 (LivaNova, Houston, TX, USA). Initial stimulation parameters were set at a current of 0.25–0.5 mA, a frequency of 20–30 Hz, a Pulse width of 250–500 μs, and a 30-s on-time and 5-min off-time. The device parameters were adjusted by the treating paediatric neurologist according to accepted guidelines during subsequent outpatient visits [10, 30].

### Statistical analysis

The SPSS (Statistical Package for the Social Sciences) version 16.0 software was used for statistical analysis. Demographic characteristics were analyzed using descriptive statistics. Chi-Square tests were used to analyse categorical data, and T-tests were used to analyse continuous variables. A p-value of < 0.05 was deemed significant. The Spearman Correlation test was used to evaluate the direction and strength of the association between the different variables. Bonferroni-adjusted pairwise comparisons were performed to assess differences in McHugh seizure outcome scores across clinically relevant follow-up duration intervals (< 18 months, 18–23 months, 24–35 months, and > 35 months).

## Results

### Study population

The sample consisted of 93 patients who underwent surgery for VNS implantation between 2008 and 2022, with an average follow-up period of 28.6 months and a minimum follow-up period of 12 months. The average age of the patients at the time of VNS insertion was 7 years old with a range from 6 months to 12 years old. Gender included 50 male patients and 43 female patients, with a similar mean age across genders (see Table 1). The average time between first seizure and VNS implantation was 61.9 months (see Table 1). The most common aetiology of epilepsy according to the ILAE classification for epilepsy and epilepsy syndromes was genetic (see Table 1), and the most common dominant seizure type according to the ILAE classification was generalized onset seizures (see Table 1). The number of antiepileptic drugs trialed prior to VNS insertion ranged between 3 to 7 over their lifetime, with an average of 4.

**Table 1:** Patient demographics

Patient demographics	*n* (%)
Number of patients	93
Sex	
Male	50 (54)
Female	43 (46)
Average age of insertion (Years)	7
Average time between insertion and seizure onset (Months)	61.9
Follow-up (months)	28.6
Aetiology of epilepsy	
Genetic	33 (35)
Immune	9 (7)
Infectious	5 (5)
Structural	14 (15)
Unknown	33 (35)
Dominant seizure type	
Generalized onset seizures	51 (55)
Focal onset, impaired awareness	23 (25)
Non-motor seizures	9 (10)
Myoclonic seizures	5 (5)
Focal onset seizures	5 (5)

The minimum number of antiepileptic medications attempted prior to VNS insertion was 3. The average number of AEDS at the time of device insertion was 4. Fifty-two patients (56%) were on or attempted and failed a ketogenic diet at some stage during treatment.

### Effect of VNS on seizure reduction

A total of 56 patients reported at least a 50% reduction in seizure frequency from baseline. Amongst the responders, the average time to response was 6.9 months (see Table 2). The average Engel and McHugh scores were 3 and 2, respectively. There were 16 (17%) McHugh class I, 37 (40%) McHugh class II, 32 (34%) McHugh class III, 2 (2%) McHugh class IV, and (6%) McHugh class V (see Table 3). There was no association between gender and response rate (*p* = 0.371). There was no association between the time to response and final Engel and McHugh scores.

**Table 2:** Outcomes of VNS

Gender of patient	Average time to respond (Months)	Pre VNS HSSS	Post VNS HSSS	Change in HSSS	Engel seizure reduction score	McHugh seizure reduction score
Male	**7,9**	**33**	**28**	**5**	**3**	**3**
Female	**5,9**	**36**	**26**	**10**	**2**	**2**
Sample	**6,9**	**34**	**27**	**7**	**3**	**2**

*HSSS* Hague Seizure Severity Score

**Table 3:** McHugh seizure frequency reduction score

Class	Description	Number (%)
I	80- 100% reduction in seizure severity	16 (17%)
II	50- 79% reduction in seizure frequency	37 (40%)
III	< 50% reduction in seizure frequency	32 (34%)
IV	Magnet benefit only	2 (2%)
V	No benefit	6 (6%)

### Effect of age on VNS response

A statistically significant association was found between age at insertion and seizure response, with younger patients exhibiting a greater response to VNS than older patients (*p* < 0.001). This was further strengthened by findings that showed a statistically significant positive association between age and Engel and McHugh scores, indicating that increased age results in increased Engel and McHugh scores (*p* = 0.047) and (*p* < 0.001), respectively. There was a statistically significant association between the duration between the first recorded seizure and McHugh scores, with results showing that the shorter the duration from the first reported seizure and device insertion, the lower the McHugh scores (Spearman’s correlation, r = −0.435, *p* < 0.001).

### Effect on seizure severity

The average Hague seizure severity score (HSSS) before and after VNS insertion were 34 and 27, respectively, with an average change in HSSS of 7 (see Table 2). There was a statistically significant association between age and HSSS post VNS insertion, with results showing the younger the age of the patient, the more likely the patient was to have a lower HSSS post insertion of VNS (*p* = 0.015); however, this did not translate to a statistically significant change in HSSS pre and post insertion (*p* = 0.744). There was no association between the change in HSSS and the duration from the first reported seizure to device insertion.

### Effect of length of follow-up and VNS response

There was a statistically significant association between the length of follow-up and McHugh seizure reduction score (Pearson Chi-square = 25.750, *p* = 0.043). However, post hoc Bonferroni-adjusted pairwise comparisons of subgroup follow-up intervals revealed no statistically significant differences between groups. There was no association between length of follow-up and response rate (*p* = 0.84), Engel seizure reduction score (*p* = 0.885), or change in HSSS (*p* = 0.908).

### Comparison of seizure control amongst the devices

The Pulse® 102 (LivaNova, Houston, TX, USA) device was implanted in 33 (35%), the AspireSR® 106 (LivaNova, Houston, TX, USA) was implanted in 41 (44%) and the Sentiva® 1000 (LivaNova, Houston, TX, USA) was implanted in 19 (20%) of patients. The Sentiva 1000 had the highest responder rate of 73.7%, followed by the AspireSR 106 at 68.3%, and then the Pulse 102 at 42.4%. No significance was found when comparing the efficacy of reducing seizure frequency and severity among the devices using the McHugh, Engel, and Hague classification systems.

### Complications and adverse effects

There was a total of 4 (4.3%) infections, with 2 (2.2%) requiring hardware removal and 2 (2.2%) requiring wound debridement and antibiotic therapy but no device explantation (see Table 4). Of the cases requiring surgical removal, one patient required removal of the IPG, while one patient required removal of the IPG and lead system. In both these cases, a new VNS device was not reinserted. One (1%) patient developed a small keloid over the surgical incision site. Short-term device-related complications included throat irritation or coughing during stimulation in 4 (4.3%) patients, and a mild shocking sensation during stimulation in 2 (2.2%) patients (see Table 4).

**Table 4:** VNS surgery and device-related complications/Adverse effects

Complications	Number (%)
1. Surgery-related	**5 (5%)**
1.1 Infections:	4 (4.3)
1.1.1 Infections requiring device removal	2 (2.2%)
1.1.2 Infections requiring antibiotics	2 (2.2%)
1.2 Keloid	1 (1%)
2. Device-related complications/Adverse effects	**6 (6.5%)**
2.1 Cough/throat irritation during stimulation	4 (4.3%)
2.2 Mild shocking sensation during stimulation	2 (2.2%)

### VNS effect on antiepileptic drugs

There was no meaningful reduction in the number of antiepileptic drugs required following VNS insertion, only 3 patients (3%) were able to reduce their number of AEDs required at the time of last follow up.

## Discussion

Vagal nerve stimulation (VNS) is a well-established adjunctive treatment option for DRE, irrespective of seizure type [6, 8, 24, 27]. In this study, we present, to our knowledge, the first retrospective study on VNS for drug-refractory epilepsy conducted in Sub-Saharan Africa.

This study confirms the efficacy and safety profile of VNS. Our results revealed that 60% of the children were responsive to VNS surgery, with a seizure reduction of at least 50% from baseline. The average McHugh and Engel scores were classes 2 and 3, respectively. This is in line with the current literature from a first-world setting, with response rates at 24 months ranging from 43 to 75% [3, 6, 16, 17, 24, 28, 29].

In this study, we found no association between gender and the efficacy of VNS for seizure reduction, which is consistent with the current literature [5, 9, 15, 23, 27]. A statistically significant association was found between age at insertion and seizure response, with results indicating that younger patients may experience a greater response to VNS compared to older patients. Our results revealed a significant association between an increasing age and higher final McHugh and Engel classes. The existing literature is divided regarding this association between age at insertion and seizure response. Three previous retrospective studies conducted in the paediatric population found a significant association with greater VNS seizure reduction in younger patients [1, 13, 17], whereas in other single-center retrospective studies no such association could be demonstrated [2, 6, 11, 16, 23, 24, 28, 29]. A meta-analysis conducted by Englot et al. revealed an increased VNS response rate in children younger than 6 years old compared to older children [7].

Our results revealed a statistically significant association between a shorter duration from the first reported seizure to device insertion and the final McHugh class, suggesting better VNS efficacy with a shorter duration from the first seizure and device insertion. This is in contrast with the existing literature, in which this association has not been clearly demonstrated [3, 6, 11, 16, 28, 29].

The average time to response in our sample was 6.7 months. We observed a statistically significant association between follow-up duration and McHugh class; Bonferroni-adjusted post hoc analyses did not identify substantial pairwise differences. This indicates the observed effect may reflect a gradual trend of improvement in response rather than a distinct improvement at specific time points. This aligns with the current literature, which suggests that response rates increase with longer treatment duration. [1, 2, 17, 24, 28]. This is apparent in our study, even after controlling for a minimum follow-up of 12 months.

In contrast, there was no statistically significant association between the duration of follow-up and the Engel outcome or Responder status. This may suggest an increased sensitivity of the McHugh Seizure Severity Score to measure seizure response due to its unique assessment of magnetic benefit and categorization of seizure reduction into percentages, when compared to the Engel and Responder status in this population. This is consistent with a 2012 study that assessed interrater variability among different seizure outcome scales in a pediatric population with VNS for drug-resistant epilepsy; they found the McHugh scale had the lowest interrater variability and recommended its use over other scoring systems in this population [19].

The results revealed a statistically significant association between age and HSSS after VNS insertion, indicating that younger patients were more likely to experience less severe symptoms after VNS insertion. There was, however, no association between age and the change in the HSSS before and after insertion. Although there are no studies that directly compare the association between age and HSSS post-VNS insertion, multiple studies have shown that VNS reduces seizure severity, seizure duration, and the post-ictal period [8, 17, 21, 24, 26, 27]. A study by Zakar et al. showed an average decrease in the HSSS of 5 points post-insertion, similar to the 7-point reduction experienced in our cohort. [31].

Although a higher responder rate was found with the Sentiva® 1000 (LivaNova, Houston, TX, USA) and the AspireSR® 106 (LivaNova, Houston, TX, USA) when compared to the Pulse® 102 (LivaNova, Houston, TX, USA) device, this was not reflected by significant changes in the McHugh and Engel scores across the devices. Likewise, there was no difference among the various devices and changes in the HSSS. This is concurrent with two previous retrospective studies by Muthiah et al. and Santhumayor et al. [16, 20] both performed in the paediatric population which also demonstrated greater responder rates when comparing the older open-loop systems versus the newer closed-loop systems. In a retrospective study by Tzadok et al. where older devices using traditional open-loop systems were replaced with newer closed-loop systems, they found these patients not only had a higher seizure response rate but also faster responder rates [25].

We experienced no major surgical complications or device-related side effects. Our infection rate of 4% is comparable to current literature with reported infection rates ranging from 2 to 10% and removal of the device being required in 1% to 8% of cases [1–3, 6, 11, 13, 16, 17, 22–24].

Likewise, our short-term device-related complications align with previously reported data. [1–3, 6, 11, 13, 16, 17, 22, 24, 27, 29, 31].

The study was conducted in a Sub-Saharan African context, in a low- and middle-income country. One of the main aims of our study was to determine if our population would respond differently to VNS as opposed to children in the Global North, due to ethnic, societal, cultural and resource availability differences. By in large our results were similar to studies from the developed world. These results may be skewed by the fact that although our unit is in a low to middle income setting, we are privileged to practice in a well-resourced academic hospital, which is not typical for our local environment. Once children have been referred to our unit, we can offer them treatment largely comparable to more resource rich countries. Our largest obstacle to treatment is in fact getting the patients referred to the unit in a timely manner. We serve a referral population of approximately 15 million children, receiving referrals from hospitals of up to 1600 km away. The significant challenges unique to our population included delay from diagnosis to referral; on average, our patients were 7 years old, and were referred 5 years after the diagnosis of DRE was made. In addition to the large population and vast referral pattern, we identified late referral was also largely due to a poor awareness of VNS as a treatment option for DRE. Likewise, there were several misconceptions by both the referring physicians and caregivers as to the nature of VNS therapy, particularly the noninvasive nature of the procedure. The study highlighted our need to better educate our referral centers as to the possibility of less invasive surgical treatment for DRE, as a non-insignificant stigma still surrounds more invasive resective type epilepsy surgery, discouraging both referring physicians and caregivers from engaging with a surgery epilepsy center.

## Limitations

The retrospective design of the study has inherent limitations. Our minimum follow-up period of 12 months may be insufficient to capture the full therapeutic benefit of VNS, particularly given that response to therapy often improves over time. Our study included heterogeneous epilepsy aetiologies and seizure types, which may confound the relationship between variables such as age, outcome, and device type. The sample size was not powered to perform robust stratified analyses.

## Conclusion

This study, the first to our knowledge, conducted in the Sub-Saharan context, demonstrated results comparable to those in the international literature. Given the high responder rate of 60% and the absence of major or long-term complications, the importance of increasing access to VNS in developing countries is highlighted. The findings emphasize the importance of early referral and implantation at a younger age. The study highlighted the need to better educate physicians and patients as to the possibility of less invasive surgical treatments for DRE. In our population significant stigma surrounds resective epilepsy surgery, discouraging both referring physicians and caregivers from engaging with a surgery epilepsy center. By educating physicians and caregivers as to the non-invasive nature and low complications associated with VNS therapy, we may be able to increase engagement and improve referral patterns in general to centralized epilepsy surgery centers.

## Data Availability

No datasets were generated or analysed during the current study.

## References

[1] Alexopoulos AV, Kotagal P, Loddenkemper T, Hammel J, Bingaman WE (2006) Long-term results with vagus nerve stimulation in children with pharmacoresistant epilepsy. Seizure 15:491–503. 10.1016/j.seizure.2006.06.002 16859931 10.1016/j.seizure.2006.06.002

[2] Arhan E, Serdaroglu A, Kurt G, Bilir E, Durdağ E, Erdem A, Aksakal FN, Ozcelik A, Baykaner K (2010) The efficacy of vagal nerve stimulation in children with pharmacoresistant epilepsy: practical experience at a Turkish tertiary referral center. Eur J Paediatr Neurol 14:334–339. 10.1016/j.ejpn.2009.09.010 19850501 10.1016/j.ejpn.2009.09.010

[3] Bodin E, Le Moing A-G, Bourel-Ponchel E, Querne L, Toussaint P, Berquin P (2016) Vagus nerve stimulation in the treatment of drug-resistant epilepsy in 29 children. Eur J Paediatr Neurol 20:346–351. 10.1016/j.ejpn.2016.01.011 26922364 10.1016/j.ejpn.2016.01.011

[4] Carpay JA, Vermuelen J, Stroink H, Brouwer OF, Peters AC, Aldenkamp AP, van Donselaar CA, Arts WF (1997) Seizure severity in children with epilepsy: a parent-completed scale compared with clinical data. Epilepsia 38:346–352. 10.1111/j.1528-1157.1997.tb01127.x 9070598 10.1111/j.1528-1157.1997.tb01127.x

[5] Datta P, Galla KM, Sajja K, Wichman C, Wang H, Madhavan D (2020) Vagus nerve stimulation with tachycardia detection provides additional seizure reduction compared to traditional vagus nerve stimulation. Epilepsy Behav 111:107280. 10.1016/j.yebeh.2020.107280 32759064 10.1016/j.yebeh.2020.107280

[6] Elliott RE, Rodgers SD, Bassani L, Morsi A, Geller EB, Carlson C, Devinsky O, Doyle WK (2011) Vagus nerve stimulation for children with treatment-resistant epilepsy: a consecutive series of 141 cases. J Neurosurg Pediatr 7:491–500. 10.3171/2011.2.PEDS10505 21529189 10.3171/2011.2.PEDS10505

[7] Engel J (1993) Surgical treatment of the epilepsies. Raven Press, New York

[8] Hajtovic S, LoPresti MA, Zhang L, Katlowitz KA, Kizek DJ, Lam S (2022) The role of vagus nerve stimulation in genetic etiologies of drug-resistant epilepsy: a meta-analysis. J Neurosurg Pediatr. 10.3171/2022.1.PEDS222 35303699 10.3171/2022.1.PEDS222

[9] Hamilton P, Soryal I, Dhahri P, Wimalachandra W, Leat A, Hughes D, Toghill N, Hodson J, Sawlani V, Hayton T, Samarasekera S, Bagary M, McCorry D, Chelvarajah R (2018) Clinical outcomes of VNS therapy with AspireSR® (including cardiac-based seizure detection) at a large complex epilepsy and surgery centre. Seizure 58:120–126. 10.1016/j.seizure.2018.03.022 29702409 10.1016/j.seizure.2018.03.022

[10] Heck C, Helmers SL, DeGiorgio CM (2002) Vagus nerve stimulation therapy, epilepsy, and device parameters: scientific basis and recommendations for use. Neurology 59:S31–S37. 10.1212/WNL.59.6_suppl_4.S31 12270966 10.1212/wnl.59.6_suppl_4.s31

[11] Kabir SMR, Rajaraman C, Rittey C, Zaki HS, Kemeny AA, McMullan J (2009) Vagus nerve stimulation in children with intractable epilepsy: indications, complications and outcome. Childs Nerv Syst 25:1097–1100. 10.1007/s00381-009-0849-z 19263056 10.1007/s00381-009-0849-z

[12] Labuschagne J, Mutyaba D, Nel J, Casieri C (2021) Intra-operative monitoring as an adjuvant to standard vagus nerve stimulation implantation. Childs Nerv Syst 37:3809–3816. 10.1007/s00381-021-05295-5 34302220 10.1007/s00381-021-05295-5

[13] Lagae L, Verstrepen A, Nada A, Van Loon J, Theys T, Ceulemans B, Jansen K (2015) Vagus nerve stimulation in children with drug-resistant epilepsy: age at implantation and shorter duration of epilepsy as predictors of better efficacy? Epileptic Disord 17:308–314. 10.1684/epd.2015.0768 26271818 10.1684/epd.2015.0768

[14] McHugh JC, Singh HW, Phillips J, Murphy K, Doherty CP, Delanty N (2007) Outcome measurement after vagal nerve stimulation therapy: proposal of a new classification. Epilepsia 48:375–378. 10.1111/j.1528-1167.2006.00931.x 17295633 10.1111/j.1528-1167.2006.00931.x

[15] Morrow JI, Bingham E, Craig JJ, Gray WJ (2000) Vagal nerve stimulation in patients with refractory epilepsy. Effect on seizure frequency, severity and quality of life. Seizure 9:442–445. 10.1053/seiz.2000.0417 10986004 10.1053/seiz.2000.0417

[16] Muthiah N, Zhang J, Remick M, Welch W, Sogawa Y, Jeong J-H, Abel TJ (2020) Efficacy of vagus nerve stimulation for drug-resistant epilepsy in children age six and younger. Epilepsy Behav 112:107373. 10.1016/j.yebeh.2020.107373 32942207 10.1016/j.yebeh.2020.107373

[17] Orosz I, McCormick D, Zamponi N, Varadkar S, Feucht M, Parain D, Griens R, Vallée L, Boon P, Rittey C, Jayewardene AK, Bunker M, Arzimanoglou A, Lagae L (2014) Vagus nerve stimulation for drug-resistant epilepsy: a European long-term study up to 24 months in 347 children. Epilepsia 55:1576–1584. 10.1111/epi.12762 25231724 10.1111/epi.12762

[18] Qiabi M, Bouthillier A, Carmant L, Nguyen DK (2011) Vagus nerve stimulation for epilepsy: the Notre-Dame hospital experience. Can J Neurol Sci 38:902–908. 10.1017/S0317167100012506 22030430 10.1017/s0317167100012506

[19] Rodgers WP, Durnford AJ, Kirkham FJ, Whitney A, Mullee MA, Gray WP (2012) Interrater reliability of Engel, International League Against Epilepsy, and McHugh seizure outcome classifications following vagus nerve stimulator implantation. J Neurosurg Pediatr 10:226–229. 10.3171/2012.6.PEDS11424 22816604 10.3171/2012.6.PEDS11424

[20] Santhumayor B, Karkare S, Kothare S, Rodgers S (2022) Evaluating vagus nerve stimulation treatment with heart rate monitoring in pediatric patients with intractable epilepsy. Childs Nerv Syst 38:547–556. 10.1007/s00381-021-05416-0 34837500 10.1007/s00381-021-05416-0

[21] Shan M, Mao H, Xie H, Gan Y, Wu D, Song J, Bai Y, Zhang J (2022) Vagus nerve stimulation for drug resistant epilepsy: clinical outcome, adverse events, and potential prognostic factors in a single center experience. J Clin Med 11:7536. 10.3390/jcm11247536 36556153 10.3390/jcm11247536 PMC9783695

[22] Smyth MD, Tubbs RS, Bebin EM, Grabb PA, Blount JP (2003) Complications of chronic vagus nerve stimulation for epilepsy in children. J Neurosurg 99:500–503. 10.3171/jns.2003.99.3.0500 12959437 10.3171/jns.2003.99.3.0500

[23] Soleman J, Knorr C, Datta AN, Strozzi S, Ramelli GP, Mariani L, Guzman R (2018) Early vagal nerve stimulator implantation in children: personal experience and review of the literature. Childs Nerv Syst 34:893–900. 10.1007/s00381-017-3694-5 29255920 10.1007/s00381-017-3694-5

[24] Thompson EM, Wozniak SE, Roberts CM, Kao A, Anderson VC, Selden NR (2012) Vagus nerve stimulation for partial and generalized epilepsy from infancy to adolescence. J Neurosurg Pediatr 10:200–205. 10.3171/2012.5.PEDS11489 22768964 10.3171/2012.5.PEDS11489

[25] Tzadok M, Harush A, Nissenkorn A, Zauberman Y, Feldman Z, Ben-Zeev B (2019) Clinical outcomes of closed-loop vagal nerve stimulation in patients with refractory epilepsy. Seizure 71:140–144. 10.1016/j.seizure.2019.07.006 31326720 10.1016/j.seizure.2019.07.006

[26] Wang X, Gao Q, Zhou J, Guan Y, Liu C, Pan W, Wang Q, Luan G, Li T (2017) The neuropsychological efficacy of vagus nerve stimulation in 56 children with catastrophic epilepsy. Neuropsychiatry 7:387–392. 10.4172/Neuropsychiatry.1000226

[27] Xie H, Ma J, Ji T, Liu Q, Cai L, Wu Y (2022) Vagus nerve stimulation in children with drug-resistant epilepsy of monogenic etiology. Front Neurol 13:951850. 10.3389/fneur.2022.951850 36119689 10.3389/fneur.2022.951850 PMC9475310

[28] Xie H, Ma J, Ji T, Liu Q, Cai L, Wu Y (2023) Efficacy of vagus nerve stimulation in 95 children of drug-resistant epilepsy with structural etiology. Epilepsy Behav 140:109107. 10.1016/j.yebeh.2023.109107 36758359 10.1016/j.yebeh.2023.109107

[29] Yalnizoglu D, Ardicli D, Bilginer B, Konuskan B, Karli Oguz K, Akalan N, Turanli G, Saygi S, Topcu M (2020) Long-term effects of vagus nerve stimulation in refractory pediatric epilepsy: a single-center experience. Epilepsy Behav 110:107147. 10.1016/j.yebeh.2020.107147 32604021 10.1016/j.yebeh.2020.107147

[30] Yu C, Ramgopal S, Libenson M, Abdelmoumen I, Powell C, Remy K, Madsen JR, Rotenberg A, Loddenkemper T (2014) Outcomes of vagal nerve stimulation in a pediatric population: a single center experience. Seizure 23:105–111. 10.1016/j.seizure.2013.10.002 24309238 10.1016/j.seizure.2013.10.002

[31] Zakar R, El Khoury J-V, Prince G, Boutros M, Yaghi C, Matar M, Abou Khaled K, Moussa R (2024) Quantifying vagus nerve stimulation outcomes in multifocal refractory epilepsy: a model across multiple surgeries. Cureus 16:e69284. 10.7759/cureus.69284 39282479 10.7759/cureus.69284 PMC11398724

